# The Addition of Microencapsulated or Nanoemulsified Bioactive Compounds Influences the Antioxidant and Antimicrobial Activities of a Fresh Cheese

**DOI:** 10.3390/molecules26082170

**Published:** 2021-04-09

**Authors:** Elizabeth Pérez-Soto, Antonio de Jesús Cenobio-Galindo, Salvador Omar Espino-Manzano, Melitón Jesús Franco-Fernández, Fanny Emma Ludeña-Urquizo, Rubén Jiménez-Alvarado, Andrea Paloma Zepeda-Velázquez, Rafael Germán Campos-Montiel

**Affiliations:** 1 Instituto de Ciencias Agropecuarias, Universidad Autónoma del Estado de Hidalgo, Av. Rancho Universitario s/n Km. 1., Tulancingo Hidalgo C.P. 43600, Mexico; epsoto@uaeh.edu.mx (E.P.-S.); antonio_cenobio@uaeh.edu.mx (A.d.J.C.-G.); mfranco@uaeh.edu.mx (M.J.F.-F.); ruben_jimenez@uaeh.edu.mx (R.J.-A.); andrea_zepeda@uaeh.edu.mx (A.P.Z.-V.); 2 Área Agroindustrial-Alimentaria, Universidad Tecnológica de Xicotepec de Juárez, Av. Universidad Tecnológica No. 1000, Tierra Negra, Xicotepec de Juárez, Puebla C.P. 73080, Mexico; drsalvadorespino2020@gmail.com; 3 Facultad de Industria Alimentarias, Universidad Nacional Agraria La Molina, Av. la Molina s/n, La Molina, Lima Apdo 12-056, Peru; fludena@lamolina.edu.pe

**Keywords:** xoconostle, microcapsules, nanoemulsion, antioxidant capacity, quality

## Abstract

The objective of this study was to compare the effects of the incorporation of microcapsules or nanoemulsions with *Opuntia* *oligacantha* on the quality of fresh cheese. Three treatments were established: Control, cheese with microcapsules (Micro), and cheese with nanoemulsion (Nano). The parameters evaluated were physicochemical (moisture, ash, fat, proteins, and pH), microbiological (mesophilic aerobic bacteria, mold–yeast, and total coliforms), functional (total phenols, flavonoids, and antioxidant capacity), and texture (hardness, elasticity, cohesion, and chewiness) during storage for 45 days at 4 °C. The results showed that adding microcapsules and nanoemulsion did not affect the physicochemical parameters of the cheese. Total coliforms decreased in all samples from the first days of storage (Control: 4.23 ± 0.12, Micro: 3.27 ± 0.02, and Nano: 2.68 ± 0.08 Log10 CFU), as well as aerobic mesophiles and mold–yeast counts. Regarding the functional properties, an increase in total phenols was observed in all treatments. The texture profile analysis showed that the addition of microcapsules and nanoemulsion influenced hardness (Control: 8.60 ± 1.12, Micro: 1.61 ± 0.31, and Nano: 3.27 ± 0.37 N). The antimicrobial effect was greater when nanoemulsions were added, while adding microcapsules influenced the antioxidant activity more positively.

## 1. Introduction

According to Vrdoljak et al. [1], soft, semi-hard, and hard cheeses can be classified in terms of their water activity (aw), NaCl concentration, and pH. Oaxaca cheese is considered soft based on this classification. Soft cheese is very popular in Latin American countries and is approximately 80% of the cheese consumed [2]. Particularly, fresh cheese is one of the most consumed cheeses in Mexico, and it is characterized as a soft cheese called “pasta filata”. Its production process begins with the acidification (pH 5.3 approximately) of raw or pasteurized cow’s milk; then, the curd is kneaded in hot water, salted, and finally, the formed paste is cut [3]. One disadvantage of fresh cheese is that the product undergoes different changes over time, such as in texture, which is attributed mainly to proteolysis or fermentation processes [4]. Due to its nutritional properties, cheese is considered a functional food, and there are reports of the benefits of its consumption, such as the contribution of proteins of high biological value and probiotics, among others [5]. In addition, it can be an excellent vehicle for the incorporation of bioactive compounds; Ribeiro et al. [6] incorporated rosemary extracts in cottage cheese, providing antioxidant properties, and Kim et al. [7] incorporated ginseng extract into quark cheese, improving its functional properties.

Antioxidant activity can be obtained from different sources [8]. The xoconostle (*Opuntia oligacantha*) is an acidic fruit native to the central region of Mexico, which has been consumed since pre-Hispanic times. There are reports of the high concentration of bioactive compounds in the fruit, mainly phenolic compounds such as rutin, ferulic acid, quercetin, and betalains [9]. In addition, the xoconostle has been used in traditional medicine to combat and prevent certain diseases such as type 2 diabetes, due to the bioactive compounds it contains [10], thus making it an interesting alternative for use in the food industry.

Encapsulation is the process whereby bioactive substances are introduced into a matrix with the aim of preventing their loss, protecting them from reaction with other compounds in food, or preventing oxidation reactions, maintaining their stability during storage, and even improving yield [11]. Microencapsulation by spray drying is an encapsulation technique designed to protect labile compounds against environmental factors that can damage them such as temperature and radiation, among others [12]. Zanoni et al. [13] encapsulated polyphenols from red chicory and red cabbage, and they found and determined that this encapsulation method keeps bioactive compounds stable, maintaining their antioxidant and color properties.

Due to the characteristics of essential oils, they are commonly used in the food industry. Orange essential oil is one of the most important, owing to its properties such as antioxidant and antimicrobial activity, given its components, mainly limonene, other terpenes, and phenolic compounds. Unfortunately, its low solubility, volatility, and instability make it a complicated essential oil to process and store; therefore, orange essential oil must be incorporated into a stable system, such as nanoemulsions, to maintain its characteristics [14,15].

Nanoemulsions are a novel method for the incorporation of bioactive compounds. They mainly consist of two phases that are immiscible with each other, and their use provides some advantages such as excellent stability over time [16]. There are reports of nanoemulsions applied to cheese; Artiga-Artigas et al. [17] developed an oregano-based nanoemulsion and used it as a coating for a reduced-fat cheese, improving its quality characteristics. On the other hand, Bedoya-Serna et al. [18] developed a nanoemulsion incorporating oregano extract, applied it on Minas Padrão cheese, and determined that the nanoemulsion had an antifungal effect, making it an alternative for cheese preservation. The objective of this study was to evaluate the effect of two types of encapsulation (xoconostle microcapsules and nanoemulsion with xoconostle and orange essential oil) on the physicochemical, microbiological, functional, and textural properties of fresh cheese during storage (45 days).

## 2. Results

### 2.1. Microcapsules and Nanoemulsion: Size and Concentration of Bioactive Compounds

The microcapsules with xoconostle had a particle size of 14.47 µm and phenols: 152 ± 2.02 mg gallic acid equivalents (GAE)/g microcapsules, flavonoids: 95.09 ± 1.67 mg quercetin equivalents (QE)/g microcapsules, 2,2-azino-bis (3-ethylbenzthiazoline-6-sulfonic acid) (ABTS): 95.09 ± 1.67 mg ascorbic acid equivalents (AAE)/g microcapsules, and 2,2-diphenyl-1-picrylhydrazyl (DPPH): 143 ± 2.50 mg AAE/g microcapsules.

The nanoemulsions with xoconostle and orange essential oil showed a drop in size of 98.4 nm and a Z potential of −105 mV, had excellent physical stability, and had phenols: 139.37 ± 12.14 mg GAE/g nanoemulsion, flavonoids: 83.25 ± 12.65 mg QE/g nanoemulsion, ABTS: 110.52 ± 18.31 mg AAE/g nanoemulsion, and DPPH: 157.85 ± 29.26 mg AAE/g nanoemulsion.

### 2.2. Physicochemical Analysis of Cheeses with Added Bioactive Compounds

The moisture results are shown in Table 1. There were no significant differences among the treatments (*p* > 0.05) (the beginning of the analysis Control: 48.11 ± 2.96%, Micro: 49.92 ± 0.75%, Nano: 49.07 ± 0.42%) until day 45, when all treatments started to lose moisture with respect to time, and the control lost the most moisture (39.47 ± 0.25%).

In the ash test (Table 1), no significant differences were observed among treatments during the entire process (*p* > 0.05). For each treatment, there were also no significant differences with respect to time.

For fat content (Table 1), among treatments, there were no significant differences (*p* > 0.05) on day 0. On day 15 and 30, there were differences between Micro and Nano (*p* < 0.05), the content of the latter being higher (27.71 ± 2.05% and 28.67 ± 0.55%). The fat values for all the treatments increased with respect to time.

The protein results are shown in Table 1. There were significant differences between Nano and the other treatments at the beginning of the analysis (*p* < 0.05), having a lower protein value (19.26 ± 1.01%). This trend continued to the end of the analysis (23.42 ± 0.39% protein). Regarding fat content, there was an increase in all treatments with respect to time, which is attributed to the loss of moisture in the cheeses.

The pH test (Table 1) shows that at the beginning of the analysis, Nano had the lowest value (5.29 ± 0.00), and all treatments showed a decrease with respect to time. It should be noted that at the end of the analysis, the treatment that had the greatest decrease in pH was the Control (4.53 ± 0.02).

### 2.3. Microbiological Analysis

For aerobic mesophilic bacteria (Table 2), it is observed that from the beginning of the analysis, there were differences among treatments (*p* < 0.05), Nano having the lowest concentration (4.01 ± 0.17 Log10 CFU/g), and showing the same behavior until the end of the test (7.27 ± 0.05 Log10 CFU/g).

For molds and yeasts, a behavior similar to that of aerobic mesophilic bacteria was observed (Table 2) with Nano having the lowest values (2.36 ± 0.51 Log10 CFU/g) from day 0; at the end of the test, Nano maintained this behavior, and the concentration of these microorganisms was lower (6.59 ± 0.08 Log10 CFU/g) compared to the Control (8.48 ± 0.06 Log10 CFU/g).

Table 2 shows the values obtained for total coliforms, and it explains the behavior of aerobic mesophilic bacteria and molds and yeasts. Nano had the lowest values from day 0 (2.68 ± 0.08 Log10 CFU/g), maintaining this behavior until the end of the analysis, and coliform growth was reduced three logarithmic units compared to the Control (5.73 ± 0.04 and 8.83 ± 0.09 Log10 CFU/g respectively).

### 2.4. Bioactive Compounds and Antioxidant Capacity

**Figure 1:**
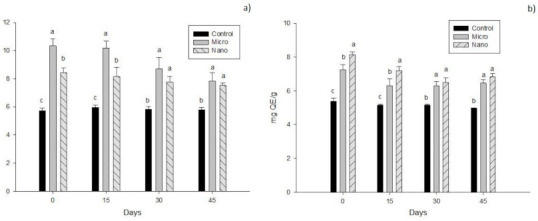
Results for total phenolic compounds (**a**) and total flavonoids (**b**) of cheeses with added bioactive compounds from xoconostle and orange essential oil. The results are expressed as means ± standard deviation. Different letters indicate significant differences (*p* < 0.05) among treatments at the same analysis day.

Figure 1a shows the results for total phenols in the different treatments. From day 0, differences were observed among treatments with Micro having the highest concentration (10.33 ± 0.50 mg GAE/g) up to day 30, after which there were no differences (*p* > 0.05) between Micro and Nano (8.69 ± 0.81 and 7.75 ± 0.43 mg GAE/g respectively). It is worth mentioning that the Control was the lowest during the entire analysis.

For total flavonoids (Figure 1b), there were differences among treatments (*p* < 0.05). As with total phenols, the Control had the lowest values, Nano being the highest on day 0 (8.15 ± 0.17 mg QE/g), and they maintained this behavior up to day 30.

**Figure 2:**
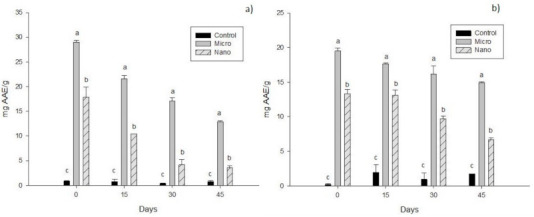
Results for antioxidant capacity by inhibiting 2,2-azino-bis (3-ethylbenzthiazoline-6-sulfonic acid) (ABTS) (**a**) and 2,2-diphenyl-1-picrylhydrazyl (DPPH) (**b**) of cheeses with added bioactive compounds from xoconostle and orange essential oil. The results are expressed as means ± standard deviation. Different letters indicate significant differences (*p* < 0.05) among treatments at the same analysis day.

The results for the inhibition of the ABTS radical are shown in Figure 2a. Micro had the highest values on day 0 (29.00 ± 0.40 mg AAE/g), followed by Nano (17.90 ± 1.97 mg AAE/g), continuing this behavior to the end of the analysis. Therefore, it can be said that microcapsules are the most efficient system for the stability of antioxidant compounds with respect to time.

Figure 2b shows that Micro maintained higher antioxidant capacity by inhibiting DPPH from day 0 (19.50 ± 0.43 mg AAE/g), as was observed for ABTS. This behavior was observed up to 45 days of the analysis.

### 2.5. Texture Profile Analysis

For hardness (Table 3), significant differences were found among treatments (*p* < 0.05). At the beginning of the analysis, the treatment that had the lowest value was Micro (1.61 ± 0.31 N), maintaining this behavior the rest of the days, while the control had the highest hardness value, which was shown from the first day (8.60 ± 1.12 N) to the end of the analysis (2.88 ± 0.80 N).

The elasticity results (Table 3) show that there were no differences among the treatments (*p* > 0.05), which indicates that the addition of bioactive compounds encapsulated by microcapsules or nanoemulsion does not affect this parameter. This is a useful characteristic for food quality.

Table 3 shows the results for cohesiveness. No significant differences were observed among the treatments (*p* > 0.05), which is a behavior similar to that observed for elasticity.

In the results of chewiness, as with elasticity and cohesiveness, there were no differences among the treatments (*p* > 0.05).

## 3. Discussion

### 3.1. Physicochemical Analysis of Cheeses Added with Bioactive Compounds

The decrease in moisture in the cheese is related to the migration of water to the surface of the matrix and its subsequent evaporation [19]. Sandoval-Copado et al. [20] determined the moisture content in different samples of fresh cheese, and they found that the values varied between 51% and 53%. Meanwhile, Colín-Cruz et al. [21] in their analysis of fresh cheese found percentages of moisture ranging from 51 to 60, which are similar to those found on day 0 in the cheeses prepared in this study. Pimentel-González et al. [11] noted that upon adding encapsulated bioactive compounds, the structural arrangement of the cheese was modified. The addition of certain ingredients may be fundamental for the moisture content. This parameter was stable in cheese with microcapsules due to the hygroscopic nature of the wall material (maltodextrin–arabic gum).

Fuentes et al. [4] determined the percentage of ash in three fresh cheeses from different processing plants and found that the ash content was between 3% and 3.8%, which is similar to the values reported in this study.

The increase in fat may be due to the composition of the nanoemulsion, which contains orange essential oil, causing a similar effect as reported by Almaráz-Buendia et al. [22]. They added nanoemulsion containing orange essential oil in sausages and observed a directly proportional increase in their fat content, since this oil is mainly comprised of terpenes. Sandoval-Copado et al. [20] recorded fat values between 20% and 22% in fresh cheese, which was lower than the values reported in this study.

Although an ingredient used as wall material (arabic gum) for the microcapsules had a small protein fraction, this did not affect the percentage of protein obtained in the cheese. Sandoval-Copado et al. [20] reported 22% protein in fresh cheese and recognized that factors such as the use of starter cultures and the processing time may increase syneresis, which would lower, resulting in higher protein content. Villanueva-Carvajal et al. [23] mentioned that protein content determined the flavor profiles of mature cheeses but did not have a considerable effect on the flavor profile of fresh cheese.

Changes in pH can be attributed to bacterial growth. According to Ramos et al. [24], a decrease in pH may be due to bacterial activity that metabolizes lactose into lactate. Caro et al. [25] found that their fresh cheese had lower pH values compared to other Mexican fresh cheeses such as panela, tenate, and morral, among others, and they mentioned that low pH and other processing steps such as kneading can cause a loss of certain soluble ions (Ca^2+^, Mg^2+^, K^+^). It should be emphasized that the addition of bioactive xoconostle compounds contained in microcapsules and nanoemulsion did not modify the physicochemical characteristics of the cheeses; hence, the product had the same characteristics as a traditional cheese.

### 3.2. Microbiological Analysis

Cheese can reach up to 9 log colony forming units (CFU)/g of aerobic mesophilic bacteria during its preparation; however, foods that contain more than 6 log CFU/g of this bacteria are prone to decomposition, which is noted by smell, taste, or appearance, thus acting as an indicator of the hygienic treatment in the process [26]. There are reports that indicate that cheese may have high concentrations of aerobic mesophilic bacteria due to its preparation. Fuentes et al. [4] analyzed commercial Oaxaca cheese samples obtained from various factories and found that the initial aerobic mesophilic bacteria concentration was 7.46 log CFU/g, which increased to 8.32 log CFU/g by day 16 of their analysis. Our results were lower than those reported by Fuentes et al. [4] by more than 2 log CFU/g for all the treatments at the beginning of our analysis. We did not obtain concentrations as high as those reported by Fuentes et al. [4] until day 40 in the controls. The differences in mesophilic bacteria concentration observed among treatments imply that the most appropriate treatment for this parameter was Nano. This effect can be attributed to the synergy between the bioactive compounds in the nanoemulsion. Although the particle size of nanoemulsions could be considered a mechanism of action when penetrating the membrane, there are reports that indicate that it does not necessarily mean an increase in the functionality of nanoemulsions, since their antimicrobial activity is due to the entire system [27]. The addition of bioactive compounds altered the concentration of mesophilic bacteria in the treatments, and there are several reports of the antimicrobial effect of xoconostle: Espinosa-Muñoz et al. [28] reported that *O. oligacantha* extracts contain phenolic compounds with an inhibitory effect on *Salmonella typhimurium* and *Staphylococcus aureus*. Furthermore, Cenobio-Galindo et al. [9] determined the antimicrobial effect of free extract and microcencapsulated extract of xoconostle added to starch films against *S. typhimurium*, and they found that the microcapsules had the greatest effect owing to the fact that the phenolic compounds attack the cell walls and membranes, thus affecting the microcapsules’ permeability and the release of intracellular constituents, causing the bacterium to die. Regarding the effect of orange essential oil and its contribution to the antimicrobial activity in this cheese, de Gomes et al. [29] mentioned that orange essential oil can be an alternative to control microorganisms, due to the components in the oil, and they stated that the mechanism of action was varied, highlighting the action on the cell wall and membrane, which interrupts ATP production and homeostasis.

As for Nano, their antimicrobial activity was against fungi and yeasts. These microorganisms are normally undesirable in soft cheeses, but to some extent, they may positively contribute to the aroma of traditional cheeses. It should be noted that fungi and yeasts may not only deteriorate characteristics of cheese but also pose a risk to public health [4,30]. Although the results show that there was an interesting effect, there are still no reports of the antifungal effect of xoconostle to corroborate these findings. Orange essential oil has been effective against these microorganisms: Viuda-Martos et al. [31] determined its effect against some microorganisms such as *Aspergillus niger* and *Penicillium chrysogenum* among others, and they determined that and determined that orange essential oil is highly effective in controlling microbial growth, attributing this effect to the components the oil contains, such as D-limonene, linalool, citral, or some phenolic compounds. Furthermore, they stated that the oil had a toxic effect on the functionality and structure of the cell membrane, causing enzymatic changes and loss of homeostasis related to energy production, among other processes. There are reports of the antifungal effect of orange essential oil incorporated into a nanoemulsion: Radi et al. [32] determined its effect as a coating applied to orange slices, which significantly reduced mold and yeast, slowing their growth for up to 17 days, and they mentioned that these systems are capable of improving the bioavailability of certain compounds such as essential oils, allowing the droplets to come in contact with a wider surface area of the microorganisms’ cell walls, thus increasing the amount of bioactive compounds that penetrate the cell. The synergistic effect of xoconostle extract and orange essential oil in the nanoemulsion cannot be ruled out.

Coliforms are related to fecal contamination and are used as a hygiene index in cheese and are considered one of the most important spoilage bacteria in fresh cheeses [4,33]. Hayek and Ibahim [34] determined the effect of xoconostle fruit against *Escherichia coli* O157: H7, noting a significant effect at concentrations of 4%, 6%, 8%, and 10% (*v*/*v*), which they attributed to compounds in the fruit, such as phenolic compounds, ascorbic acid, and betalains, mentioning that bioactive compounds have the ability to alter the cell wall permeability, causing the cell to lose macromolecules. Another mechanism is that phenolic compounds interfere with membrane function by interacting with membrane proteins, causing deformations in the structure and functionality, suggesting synergistic activity among all the components instead of just a single component’s activity. The efficacy of orange essential oil against coliforms has been evaluated: Alparslan et al. [35] examined the effect of gelatin-based coatings that contained orange essential oil on pink shrimp, and they determined that the antimicrobial effect was influenced by the amount of oil added to the coatings, achieving up to 85% more inhibition of coliforms compared to films without the oil.

The effectiveness of incorporating bioactive compounds contained in microcapsules in cheese has already been reported by Fernandes et al. [36]. They made microcapsules containing rosemary essential oil, which they applied to Minas Frescal cheese, and they found that the cheese containing the microcapsules had less microbial growth throughout the analysis, without altering the characteristics of the cheese. Just as there are reports of the efficacy of microencapsulation, there is research related to the incorporation of nanoemulsions in cheeses. Artiga-Artigas et al. [17] applied a nanoemulsion as a coating to low-fat cheese, observing that the nanoemulsion turned out to be an effective barrier to maintain the characteristics of the cheese by reducing the growth of *Staphylococcus aureus*, psychrophiles, and molds and yeasts for up to 24 days. Nanoemulsions containing oregano essential oil were developed by Bedoya-Serna et al. [18], which were tested in Minas Padrão cheese, and they determined that the nanoemulsion had an inhibitory effect on *Cladosporium* sp., *Fusarium* sp., and *Penicillium* sp., noting that it could become an alternative for the conservation of this cheese. The main objective of the application of encapsulated bioactive compounds is to improve the characteristics of cheeses during storage, maintaining their effectiveness, without compromising their physicochemical properties.

### 3.3. Bioactive Compounds and Antioxidant Capacity

One hypothesis of the differences in total phenols is that Micro has a higher concentration of these compounds due to the formulation of the microcapsules. This is because the xoconostle has a high concentration of phenols, which are protected by the wall material used in encapsulation, unlike the nanoemulsions that had less xoconostle added to them and are mainly composed of non-phenolic bioactive compounds. Several reports have suggested that xoconostle is an excellent source of phenolic compounds. Osorio-Esquivel et al. [37] evaluated these compounds in xoconostle and determined that the highest concentration of phenolic compounds was found in the pericarp, explaining that they serve as a defense to the plant against ultraviolet light and against pathogenic microorganisms; and they are rich in acids such as syringic, vanillic, caffeic, 4-hydroxybenzoic, and protocatechuic, in addition to flavonoids. Orange essential oil also contains some phenolic compounds: Khan et al. [38] found the total phenolic compounds in orange essential oil to be between 6 and 7 mg/g. There are reports of the incorporation of phenolic compounds in cheese: Deolindo et al. [39] incorporated phenolic compounds from *Vitis labrusca* to Petit Suisse cheese, and they observed that phenolic compounds decreased during storage, probably being affected by light, temperature, pH, or enzymes. Jansen-Alves et al. [40] made microcapsules containing propolis extract and added them to Minas Frescal cheese, evaluating phenolic compounds after in vitro digestion, and they determined that the bioactive compounds were stable and their release varied depending on the wall material, making it an excellent option to stably incorporate phenolic compounds in a complex food matrix. The incorporation of phenolic compounds contained in a nanoemulsion added to cheese has not been reported.

The increase in total flavonoids in Nano may be due to the fact that both sources of bioactive compounds used in the production of the nanoemulsion (xoconostle and orange essential oil) contain flavonoids. Some flavonoids in the essential oil were determined by Tenorio Dominguez [41], who discovered that they were mainly naringin, hesperidin, neohesperidin, and rutin, which have been linked to a certain suppressing of carcinogenesis. Cortez-García et al. [42] reported the presence of several flavonoids in xoconostle, highlighting quercitrin, catechin, and kaempferol, among others, and they mentioned that the interest in phenolic compounds is due to the potential relationship between their consumption and the prevention of various diseases.

The xoconostle fruit has been proven to have excellent antioxidant capacity. Cortez-García et al. [42] determined antioxidant activity by inhibiting ABTS to fresh and cooked xoconostle, and they determined that the fresh fruit had greater capacity, followed by microwaved, steamed, boiled, and finally grilled xoconostle, demonstrating that the antioxidant capacity is not totally lost after subjecting the antioxidant compounds of the fruit to heat treatments, as occurred in this study when the curds with bioactive compounds were subjected to a stretching process in hot water (75 °C). Tenorio Dominguez [41] evaluated the antioxidant capacity of orange essential oil, which showed excellent free radical uptake (greater than 90%), attributing to its main components, terpenes and metabolites, such as flavonoids, vitamin C, or carotenoids, highlighting their synergistic effect.

The difference in antioxidant activity may be because the bioactive compounds in Micro are protected by the maltodextrin–arabic gum matrix, while the compounds that form the oily phase (major component) of the nanoemulsion are in direct contact with the food matrix, decreasing the antioxidant activity potential of all the components, thus making the microcapsules a better alternative to maintain the antioxidant activity. There is much interest in the antioxidant activity of cheese. Revilla et al. [43] examined the antioxidant capacity of several cheeses, varying the types of milk, preparation periods and storage, and they determined that, as expected, the results were very varied: antioxidant activity increased with respect to time, which was attributed to a progressive proteolysis, generating soluble peptides and sulfur amino acids whose antioxidant capacity is well known. Ribeiro et al. [6] determined the antioxidant activity of extracts from two fungal species added in free and encapsulated to cottage cheese, which resulted in products with an antioxidant capacity superior to the control from the beginning of their analysis until day 7. They mentioned that the best activity in cheeses with added microcapsules can be explained by the effective protection provided by the spray-drying process together with a controlled release. Meanwhile, Deolindo et al. [39] determined the antioxidant activity in *Petit Suisse* cheese with added antioxidants from Bordeaux grape, and they found that the analyzed control and the commercial cheese did not have detectable antioxidant capacity, while the cheeses with added grape extract had a high capacity, which decreased with respect to time, and it directly affects the importance of the encapsulation of the bioactive compounds. According to what was observed for total phenols, total flavonoids, ABTS, and DPPH, the bioactive compounds added in the cheeses are maintained after processing and storage, compared to the control, giving the traditional fresh cheese functionality, since these compounds provide multiple benefits to food stability and humans.

### 3.4. Texture Profile Analysis

Regarding the decrease in hardness, Colín-Cruz et al. [21] mentioned that while the curd is kneaded and stretched, the proteins continue to interact until the fat hinders this interaction, forming cavities between the protein fibers (fat–whey channels) where the fat and the aqueous phase are retained. Therefore, variations in the content of ingredients affected the microstructure, which was probably because the microcapsules or the nanoemulsion were trapped in these cavities, directly impacting the hardness. A hypothesis related to the previous assumption is that the microcapsules, which are larger than the nanoemulsions, significantly affected the cheese’s microstructure and did not develop a homogeneous network between the components. Furthermore, the release of microcapsules during storage affected the results even more, simultaneously decreasing with respect to time, showing the same behavior as reported by Kim et al. [44].

Sandoval-Copado et al. [20] analyzed the texture of fresh cheese when the form of acidification of milk was modified, and they determined that there were no significant differences in elasticity among their treatments, mentioning that this is related to agitation and moisture content. Stirring the curd for long causes more whey to be expelled, and the cheese has a lower percentage of moisture, with greater interaction between proteins, making the cheese firmer and more compact.

Fuentes et al. [4] determined the cohesiveness of fresh cheese and found that there were no differences with respect to time (24 days of storage), with values lower than those reported in this work. Likewise, Caro et al. [3], when making fresh cheese and adding skim milk and milk protein concentrate during production, did not observe differences in cohesiveness.

Sandoval-Copado et al. [20] noted that there was an inversely proportional relationship between the chewiness and moisture of fresh cheese. Artiga-Artigas et al. [17] examined the texture profile in reduced-fat cheese slices coated with a nanoemulsion containing oregano essential oil and tangerine fiber, and they determined that the slices that were not coated had higher values in all the parameters evaluated, mentioning that the coated cheese was less hard. Pimentel-González et al. [11] evaluated the effect of the addition of grape phenolic compounds in Chihuahua cheese, and they determined that the hardness increased during storage at 4 °C, which is associated with a loss of moisture, while the elasticity and cohesiveness depended on the concentration of polyphenols added, but no differences in chewiness were observed. In this study, the texture profile analysis revealed that the addition of xoconostle bioactive compounds to microcapsules and nanoemulsion affected the hardness but not the elasticity, cohesiveness, and chewiness; therefore, a great part of the quality of the product is not compromised.

## 4. Materials and Methods

### 4.1. Materials

The materials used in this study were ethanol (HPLC grade), maltodextrin (Grain Processing Corporation, Muscatine, Schaumburg, IL USA), arabic gum (Frutarom, Schaumburg, IL, USA), orange essential oil (Sigma-Aldrich, St. Louis, MO, USA), soy lecithin (Sigma-Aldrich, USA), citric acid (Sigma-Aldrich, USA), calcium chloride (J.T. Baker, Phillipsburg, NJ, USA), commercial rennet (Christian Hansen, Mexico City, Mexico), tryptone–yeast extract agar (Sigma-Aldrich, USA), red bile violet agar (Sigma-Aldrich, USA), potato dextrose agar (Sigma-Aldrich, USA), Folin–Ciocalteau reagent (Sigma-Aldrich, USA), sodium carbonate (J.T. Baker, USA), sodium nitrite (J.T. Baker, USA), aluminum trichloride (Sigma-Aldrich, USA), sodium hydroxide (J.T. Baker, USA), 2,2-diphenyl-1-picrylhydrazyl (DPPH, Sigma-Aldrich, USA), methanol (analytical reagent grade), 2,2-azino-bis (3-ethylbenzthiazoline-6-sulfonic acid) (ABTS, Sigma-Aldrich, USA), and potassium persulfate (analytical reagent grade).

### 4.2. Extraction of Bioactive Compounds from the Xoconostle Fruit

The extraction of bioactive compounds from xoconostle was carried out following the methodology by Espinosa-Muñoz et al. [28]. Manually selected *Opuntia oligacantha* fruits free from physical damage were obtained from the municipality of Tetepango, Hidalgo, Mexico (20°06′11″ N, 99°09′23″ W); they were cut into thin slices and subjected to freeze-drying for two days using a Freeze Equipment Dry System (Labconco, Kansas City, MO, USA). After that time, the product obtained was pulverized and subjected to ultrasound with a 50% ethanol solution, using a Vibra-cell VCX130 equipment (Sonics, Newtown, CT, USA), the extraction protocol was 20 min, 130 W, 80% amplitude, and a frequency of 20 kHz. Finally, the extract was filtered (Whatman No. 1 paper) and centrifuged at 10,000 rpm for 15 min at 4 °C using a Z36HK centrifuge (Hermle, Gosheim, Germany). The samples were stored in complete darkness until use.

### 4.3. Preparation of the Microcapsules

The microcapsules were made following the methodology by Cenobio-Galindo et al. [9]. The xoconostle extract (4% of total solids) was mixed with two polymers that served as wall material, maltodextrin and arabic gum (in equal proportions), until making up 30% of solids in the mixture, keeping it in agitation and darkness for 24 h. The homogeneous mixture was dried using a Mini Spray Dryer (Büchi B-290, Flawil, Switzerland) at 160 °C, 4 bars of pressure, and an inlet flow of 10 mL/min, the microcapsules were stored in complete darkness at 4 °C until use. To determine the particle size of the microcapsules, the methodology by Pérez-Alonso et al. [45] was used.

### 4.4. Preparation of the Nanoemulsion

The nanoemulsion was prepared following the methodology by Espino-Manzano et al. [46]. The continuous phase (70%) was orange essential oil (Sigma-Aldrich, USA), the dispersed phase was xoconostle extract (20%) and soy lecithin (Sigma-Aldrich, USA) as surfactant (10%). A Vibra-Cell VCX 130 ultrasonic processor (Ultrasonic, Newtown, CT, USA) was used, with a 6 mm probe, the sonication conditions were 20 one-minute intervals, rest periods of 10 s, 80% amplitude and frequency of 20 kHz. The dispersion obtained was stored in the darkness at 4 °C. To determine the particle size and the z potential of the nanoemulsion, the methodology by Cenobio-Galindo et al. [47] was used.

### 4.5. Manufacture of Cheeses Added with Bioactive Compounds

The cheeses were made following the methodology by Rodríguez-Huezo et al. [48], with some modifications. First, 15 L batches of raw milk (135 g/L of total solids, 35 g/L of milk fat, 34 g/L of protein) were provided by the company PROUNILAC (Tulancingo de Bravo, Hidalgo, Mexico; 20°05′09″ N, 98°21′48″ W) and were standardized with skim milk to up to 32 g/L of milk fat. The standardized batches of milk were pasteurized in a tub at 72 °C for 15 min. The milk was cooled to 36 °C, and citric acid (10% *w*/*w*) was added until a pH of 5.47 ± 0.03 was reached; subsequently, calcium chloride (0.20 g/L) and commercial rennet (0.12 mL/L; Christian Hansen, Mexico) were added. At this point, the encapsulated bioactive compounds (50 g/L of microcapsules or nanoemulsion; value determined in relation to the similarity in quantification of bioactive compounds of both encapsulates) were added to the milk batches. After 30 min, the curd was cut into 1 cm^3^ pieces, and 80% of the whey was drained; then, it was slowly stirred for a further 30 min. The curd was manually stretched to 6 cm wide in hot water (75 °C), and a smooth, elastic paste was formed. The strips were immersed in water at 20 °C, 1 g of NaCl was added to them, the cheese formed was placed in a hermetically sealed plastic bag and stored at 4 °C, until analysis. In this study, three treatments were developed; cheese without the addition of encapsulated bioactive compounds (Control), cheese with added microcapsules (Micro), and cheese with added nanoemulsion (Nano), and they were evaluated at days 0, 15, 30, and 45 of storage. Each treatment was evaluated in all tests in triplicate and to ensure repeatability. All analyses described below were determined twice.

### 4.6. Determination of Physicochemical Parameters

For the physicochemical parameters in cheeses, the following were evaluated in reference to the Association of Official Analytical Chemists (AOAC) [49]: moisture (method 926.08), ash (method 935.42), fat (method 933.05), and protein (method 920.123), and pH was determined by [10].

### 4.7. Microbiological Analysis

The microbiological analyses were carried out following the methodologies described by Ashenafi [50] with certain modifications. First, 10 g of cheese were added to 90 mL of peptone solution; then, they were homogenized for one minute, and from this, consecutive dilutions were made in 0.9% NaCl solution. The analysis comprised of the counts of aerobic mesophilic bacteria, in tryptone–yeast extract agar at 35 °C, total coliforms in red bile violet agar incubated at 35 °C for 24 h, and molds and yeasts in potato dextrose agar at 28 °C for 72 h. After the respective time, the microbial counts were made, and the results are expressed in Log_10_ CFU/g.

### 4.8. Extraction of Bioactive Compounds

For the extraction of phenolic compounds and antioxidant activity, the methodology described by Deolindo et al. [39] was used with some modifications. First, 10 g of cheese was mixed with 90 mL of methanol at 25 °C; then, the mixture was crushed and centrifuged at 10,000 rpm. Then, the supernatant was used to determine total phenols, total flavonoids, ABTS, and DPPH (Section 4.5, Section 4.6, Section 4.7 and Section 4.8).

### 4.9. Determination of Total Phenols

The total phenolic content was determined by the Folin–Ciocalteu test described by Pimentel-González et al. [11], with some modifications. First, 1 mL of sample was mixed with 5 mL of diluted Folin–Ciocalteau reagent (1:10). After 6 min, 4 mL of sodium carbonate (20%) was added to the mixture, and it was allowed to stand for 2 h at room temperature, and the absorbance at 760 nm was determined with a 6715 UV-Vis spectrophotometer. The total phenolic content was expressed as mg gallic acid equivalents/g of cheese. The analysis was carried out in triplicate.

### 4.10. Determination of Total Flavonoids

The total flavonoid content was measured using the method described by Munir et al. [51] with some modifications. First, 1 mL of sample was mixed with 4 mL of deionized water in a 10 mL flask. Then, 300 µL of 5% sodium nitrite was added to the flask, and after five minutes, 300 µL of 10% aluminum trichloride was also added. Then, 2 mL of 1 M sodium hydroxide were added to the mixture, which was diluted to 10 mL with deionized water. The solution was mixed, and the absorbance at 415 nm was measured. Total flavonoid content was expressed in mg quercetin equivalents/g cheese. The analysis was carried out in triplicate.

### 4.11. Evaluation of Antioxidant Capacity by Inhibition of 2,2-diphenyl-1-picrylhydrazyl (DPPH)

First, a solution of 6.5 × 10^−5^ M DPPH in 80% methanol was prepared and stirred continuously for 2 h in the darkness. Then, 0.5 mL of sample was mixed with 2.5 mL of the DPPH solution, and the mixture was stirred. The mixture was allowed to react for 1 h, and the final absorbance was read on a 6715 UV-Vis spectrophotometer. The results obtained were expressed in mg ascorbic acid equivalents/g of cheese [11]. The analysis was carried out in triplicate.

### 4.12. Evaluation of the Antioxidant Capacity by the Inhibition of 2,2-Azino-bis (3-ethylbenzthiazoline-6-sulfonic acid) (ABTS)

The determination of the antioxidant activity by means of ABTS radical inhibition was carried out as described by Pimentel-González et al. [11] with some modifications. A 10 mL solution of 7 mM ABTS was prepared and reacted with 10 mL of 2.45 mM potassium persulfate. The mixture was stirred for 16 h in a container in complete darkness. Then, 200 µL of the sample extract was added to 2 mL of ABTS solution and reacted for 6 min; subsequently, the absorbance was read at 734 nm. The results were expressed in mg ascorbic acid equivalents/g of cheese. The analysis was carried out in triplicate.

### 4.13. Texture Profile Analysis

The test was carried out following the methodology described by Pimentel-González et al. [11], with some modifications. First, 2 cm cubes of cheese were used to test on a CT3 texture analyzer (Brookfield, United Kingdom) with a 50 kg load cell. Then, the samples were compressed using a double compression speed of 10 mm/s, compressing them to 50% of their height. The values of hardness (N), elasticity (dimensionless), cohesiveness (dimensionless), and chewiness (N) were calculated by the equipment software.

### 4.14. Statistical Analysis

An ANOVA test was performed, and when there were significant differences among the treatments, the Tukey mean comparison test (*p* < 0.05) was applied, using the IBM SPSS Statistics 20 software.

## 5. Conclusions

The addition of bioactive compounds to fresh cheese is an effective alternative to maintain and/or improve the characteristics of this food during storage and thus achieve the development of a functional food. The physicochemical properties were not affected by the addition of such compounds; however, in the microbiological analyses, a considerable decrease was observed for aerobic mesophilic bacteria, molds, and yeasts and coliforms, which is due to the compounds incorporated by both encapsulation methods, Nano being more effective. In antioxidant activity, both treatments were better than the control, but Micro had a greater capacity with respect to time. In the texture profile analysis, the hardness was affected by the addition of the bioactive compounds, but there was no effect on elasticity, firmness, and chewiness. With these results obtained from the comparison of the different encapsulates added to a traditional Mexican fresh cheese, relevant information was collected that will open large areas of opportunity for future studies. It is concluded that each encapsulation method benefited the cheese without it undergoing major changes that would affect its quality with respect to time.

## Figures and Tables

**Table 1:** Physicochemical results of the cheeses with added bioactive compounds from xoconostle and orange essential oil.

Days/Treatments	Control	Micro	Nano
**Moisture (%)**			
0	48.11 ± 2.96 ^aA^	49.92 ± 0.75 ^aA^	49.07 ± 0.42 ^aA^
15	44.43 ± 2.68 ^aB^	47.42 ± 1.74 ^aAB^	46.34 ± 1.52 ^aB^
30	42.28 ± 1.58 ^aB^	44.06 ± 3.06 ^aB^	44.41 ± 0.51 ^aBC^
45	39.47 ± 0.25 ^bC^	43.59 ± 1.43 ^aA^	42.74 ± 1.31 ^aC^
**Ash (%)**			
0	3.15 ± 0.34 ^aA^	3.44 ± 0.17 ^aA^	3.27 ± 0.08 ^aB^
15	3.37 ± 0.07 ^aA^	3.64 ± 0.35 ^aA^	3.53 ± 0.32 ^aAB^
30	3.52 ± 0.29 ^aA^	3.70 ± 0.16 ^aA^	3.60 ± 0.20 ^aA^
45	3.55 ± 0.14 ^aA^	3.72 ± 0.06 ^aA^	3.62 ± 0.11 ^aA^
**Fat (%)**			
0	24.66 ± 0.57 ^aB^	22.43 ± 1.92 ^aB^	25.33 ± 1.52 ^aB^
15	26.33 ± 1.52 ^abAB^	24.67 ± 1.15 ^bAB^	27.71 ± 2.05 ^aAB^
30	27.66 ± 1.18 ^abA^	25.60 ± 1.52 ^bA^	28.67 ± 0.55 ^aA^
45	28.31 ± 1.04 ^aA^	26.33 ± 1.14 ^aA^	29.33 ± 2.08 ^aA^
**Protein (%)**			
0	22.65 ± 0.89 ^aB^	22.49 ± 0.51 ^aC^	19.26 ± 1.01 ^bC^
15	24.03 ± 0.51 ^aB^	23.65 ± 0.09 ^aB^	21.03 ± 0.56 ^bB^
30	24.71 ± 0.55 ^aA^	24.21 ± 1.03 ^aA^	22.60 ± 0.42 ^bA^
45	25.00 ± 0.31 ^aA^	25.43 ± 0.56 ^aA^	23.42 ± 0.39 ^bA^
**pH**			
0	5.52 ± 0.05 ^aA^	5.57 ± 0.01 ^aA^	5.29 ± 0.00 ^bA^
15	5.19 ± 0.01 ^abB^	5.23 ± 0.06 ^bB^	5.13 ± 0.08 ^aB^
30	4.98 ± 0.03 ^aC^	5.02 ± 0.14 ^aB^	5.05 ± 0.09 ^aBC^
45	4.53 ± 0.02 ^cD^	4.71 ± 0.02 ^bC^	4.93 ± 0.04 ^aC^

The results are expressed as means ± standard deviation. Lowercase letters in the same row indicate significant differences (*p* < 0.05) among treatments on the same analysis day. Different capital letters in the same column indicate significant differences (*p* < 0.05) in each treatment on different analysis days.

**Table 2:** Microbiological analysis of the cheeses with added bioactive compounds from xoconostle and orange essential oil.

Days/Treatments	Control	Micro	Nano
**Aerobic mesophilic bacteria**			
0	4.88 ± 0.06 ^bA^	5.04 ± 0.15 ^bA^	4.01 ± 0.17 ^aA^
15	5.84 ± 0.03 ^cB^	5.58 ± 0.01 ^bB^	5.08 ± 0.02 ^aB^
30	7.84 ± 0.06 ^cC^	7.27 ± 0.08 ^bC^	6.36 ± 0.01 ^aC^
45	8.45 ± 0.01 ^cD^	7.64 ± 0.09 ^bD^	7.27 ± 0.05 ^aD^
**Molds and yeasts**			
0	2.60 ± 0.11 ^aA^	2.89 ± 0.22 ^aA^	2.36 ± 0.51 ^aA^
15	4.72 ± 0.05 ^cB^	3.47 ± 0.04 ^bB^	3.01 ± 0.06 ^aB^
30	7.86 ± 0.18 ^cC^	5.99 ± 0.10 ^bC^	5.49 ± 0.28 ^aC^
45	8.48 ± 0.06 ^cD^	7.91 ± 0.03 ^bD^	6.59 ± 0.08 ^aD^
**Total coliforms**			
0	4.23 ± 0.12 ^cA^	3.27 ± 0.02 ^bA^	2.68 ± 0.08 ^aA^
15	4.58 ± 0.15 ^cB^	3.54 ± 0.03 ^bB^	3.59 ± 0.01 ^aB^
30	6.30 ± 0.09 ^cC^	5.47 ± 0.03 ^bC^	4.64 ± 0.02 ^aC^
45	8.83 ± 0.09 ^cD^	6.79 ± 0.08 ^bD^	5.73 ± 0.04 ^aD^

The results are expressed in Log10 CFU/g, and are expressed as means ± standard deviation. Lowercase letters in the same row indicate significant differences (*p* < 0.05) among treatments on the same analysis day. Different capital letters in the same column indicate significant differences (*p* < 0.05) among each treatment on different analysis days.

**Table 3:** Texture profile analysis of cheeses added with bioactive compounds from xoconostle and orange essential oil.

Days/Treatments	Control	Micro	Nano
**Hardness (N)**			
0	8.60 ± 1.12 ^aA^	1.61 ± 0.31 ^cA^	3.27 ± 0.37 ^bA^
15	6.90 ± 0.34 ^aB^	0.98 ± 0.12 ^cB^	3.64 ± 0.18 ^bA^
30	5.02 ± 0.77 ^aC^	1.23 ± 0.33 ^cB^	1.78 ± 0.12 ^bB^
45	2.88 ± 0.80 ^aD^	0.83 ± 0.27 ^cB^	1.62 ± 0.17 ^bB^
**Elasticity (Adimensional)**			
0	0.75 ± 0.05 ^aA^	0.72 ± 0.05 ^aA^	0.75 ± 0.05 ^aA^
15	0.80 ± 0.08 ^aA^	0.85 ± 0.05 ^aA^	0.75 ± 0.05 ^aA^
30	0.75 ± 0.11 ^aA^	0.85 ± 0.17 ^aA^	0.81 ± 0.14 ^aA^
45	0.72 ± 0.05 ^aA^	0.72 ± 0.09 ^aA^	0.77 ± 0.09 ^aA^
**Cohesiveness (Adimensional)**			
0	0.50 ± 0.02 ^aA^	0.56 ± 0.07 ^aA^	0.51 ± 0.04 ^aA^
15	0.54 ± 0.09 ^aA^	0.52 ± 0.01 ^aA^	0.55 ± 0.02 ^aA^
30	0.55 ± 0.02 ^aA^	0.49 ± 0.03 ^bA^	0.46 ± 0.07 ^bA^
45	0.55 ± 0.03 ^aA^	0.50 ± 0.04 ^aA^	0.51 ± 0.08 ^aA^
**Chewiness (N)**			
0	6.16 ± 1.9 ^aA^	5.12 ± 1.58 ^aA^	5.11 ± 0.85 ^bA^
15	5.38 ± 0.68 ^aA^	5.20 ± 0.89 ^aA^	5.37 ± 0.29 ^aA^
30	5.77 ± 0.77 ^aA^	5.87 ± 2.07 ^aA^	5.84 ± 2.56 ^aA^
45	6.02 ± 0.33 ^bA^	5.03 ± 1.33 ^aA^	4.92 ± 1.29 ^aA^

The results are expressed as means ± standard deviation. Lowercase letters in the same row indicate significant differences (*p* < 0.05) among treatments on the same analysis day. Different capital letters in the same column indicate significant differences (*p* < 0.05) among each treatment on different analysis days.

## Data Availability

Not applicable.
